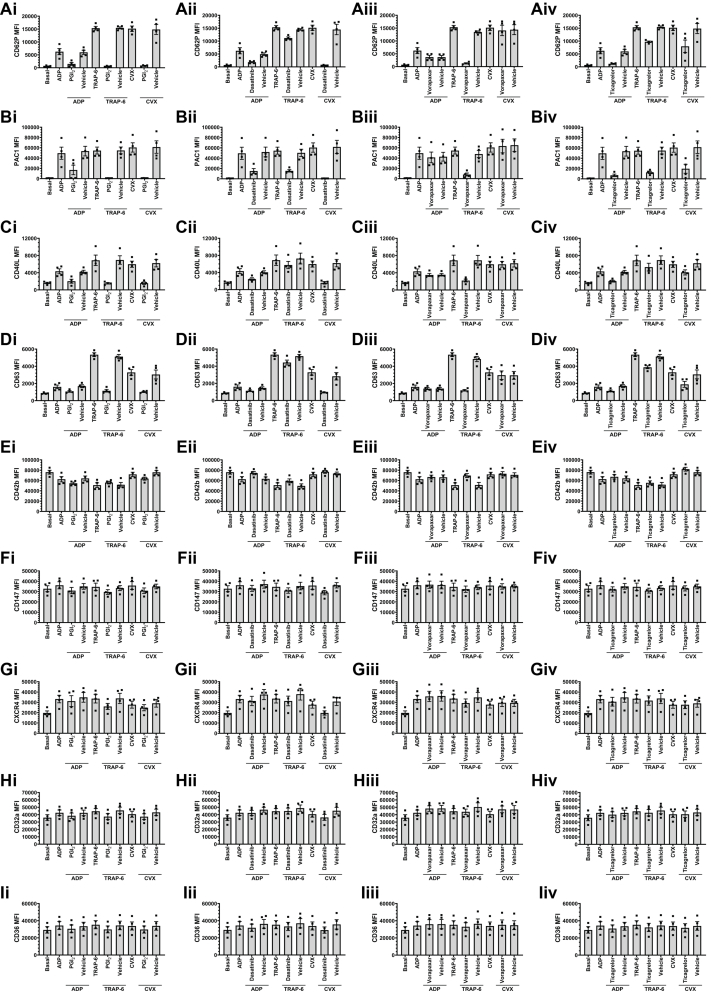# Phenotyping of human platelets in response to platelet agonists and inhibitors using multiparameter flow cytometry and unbiased high-dimensional analysis

**DOI:** 10.1016/j.rpth.2025.103189

**Published:** 2025-09-23

**Authors:** Daisie M. Yates, Benjamin E.J. Spurgeon, Lih T. Cheah, James I. Robinson, Ann W. Morgan, Khalid M. Naseem

**Affiliations:** 1 Leeds Institute of Cardiovascular and Metabolic Medicine, Faculty of Medicine and Health, University of Leeds, Leeds, United Kingdom; 2 Versiti Blood Center of Wisconsin, Milwaukee, Wisconsin, USA; 3 National Institute for Health and Care Research Leeds Biomedical Research Centre, Leeds Teaching Hospitals National Health Service Trust, Leeds, United Kingdom

**Keywords:** flow cytometry, machine learning, platelets, platelet inhibitors, platelet activation

## Abstract

**Background:**

Despite increasing evidence of functionally distinct platelet subpopulations, the role of receptor remodeling in this process is unclear.

**Objectives:**

To develop a high-dimensional multiparameter flow cytometry panel for platelet phenotyping and subpopulation analysis.

**Methods:**

Using conventional flow cytometry, we assessed the expression of 9 platelet surface receptors in whole blood (WB) samples from healthy adults in response to different platelet activators and inhibitors. Platelet subpopulations were identified by Full Annotation Using Shape-constrained Trees (FAUST).

**Results:**

The 9-parameter assay simultaneously measured the activation of α_IIb_β_3_ (defined by PAC1 antibody binding) and expression of CD62P, CD40L, CD63, CD42b, CD147, CXCR4, CD32a, and CD36. After WB stimulation with adenosine diphosphate, thrombin receptor-activating peptide-6, or convulxin, FAUST identified 8 platelet subpopulations that were defined by differential levels of PAC1, CD62P, and CD63. To determine how inhibitors affect platelet subpopulations, WB was pretreated with prostacyclin, dasatinib, vorapaxar, or ticagrelor prior to stimulation with adenosine diphosphate, thrombin receptor-activating peptide-6, or convulxin. Again, FAUST identified 8 distinct platelet subpopulations, which were defined by differential levels of PAC1, CD40L, and CD62P, showing that under conditions of platelet inhibition, CD40L expression is important for subpopulation annotation.

**Conclusion:**

Platelet subpopulations are dynamic and undergo remodeling in response to different agonists and inhibitors. This high-dimensional single-cell flow cytometry assay is a practicable approach to evaluate the effects of agonists and inhibitors on platelet receptor expression and platelet subpopulations.

## Essentials

• Remodeling of platelet surface receptors may facilitate hemostasis and innate immune responses.
• High-dimensional flow cytometry enabled the study of receptor remodeling within platelet subpopulations.
• Platelet subpopulations were determined by unique platelet surface receptor expression patterns.
• Platelet subpopulations are dynamic in response to platelet agonists and inhibitors.

## 1 Introduction

Blood platelets play key roles in hemostasis, thrombosis, angiogenesis, wound healing, and inflammation [1]. Their ability to play such varied physiological and pathophysiological roles is facilitated by an armory of surface receptors that are remodeled upon exposure to various agonists and primers found within the circulatory system [1]. Many of these receptors are constitutively expressed and are elevated in response to specific agonists, while some are translocated to the plasma membrane upon platelet activation. Distinct receptor expression in response to physiological or pathophysiological mediators may lay the basis of functional heterogeneity, along with platelet size and age, which may dictate responsiveness to activation within a given platelet population [2]. This has led to platelet subpopulation characterization, which has been facilitated by high-dimensional flow cytometry, a method that measures many parameters simultaneously on individual cells and generates enormous amounts of complex data that require advanced algorithmic tools for interpretation [[2], [3], [4]]. While the use of mass cytometry [5] and spectral flow cytometry [6,7] has emerged as techniques for deep platelet phenotyping, they remain beyond the reach of some research laboratories. Conventional flow cytometry is still the most widely used technology for single-cell analysis of multiple surface receptors and has been used to examine platelet sensitivity to activators and inhibitors [3,4,8], analyze platelets in disease settings [[9], [10], [11]], diagnose inherited bleeding disorders [12,13], and characterize platelets during the aging process [3]. At present, the information gathered through existing conventional flow cytometry panels is limited, and given the variety of receptors on the platelet surface, our current ability to discover potentially unique and rare platelet subpopulations is restricted. Therefore, we developed a novel 9-parameter flow cytometry panel that measures both activation-induced (CD62P, α_IIb_β_3_ activation, CD40L, and CD63) and constitutive (CD42b, CD147, CXCR4, CD32a, and CD36) platelet surface markers in whole blood (WB).

Activated platelets undergo α_IIb_β_3_ inside-out signaling, driving activation of the receptor and subsequent platelet aggregation in hemostasis and thrombosis due to fibrinogen binding [1,14]. This activation can be measured using the monoclonal antibody clone PAC1 [15]. Activated platelets secrete CD40L and CD62P from α-granules [16,17] and CD63 from dense granules [18] and lysosomes [19], resulting in receptor translocation to the platelet surface. CD62P and CD40L drive platelet-leukocyte aggregate formation [20,21] by binding to P-selectin glycoprotein ligand 1 and CD40 on leukocytes, respectively, enabling platelet-leukocyte aggregate tethering and rolling on the endothelium [22,23], monocyte cytokine release [24,25], tissue factor expression [26], and coagulation factor binding [27], resulting in local activation of the coagulation pathway. During hemostasis, CD42b binds von Willebrand factor, which simultaneously binds subendothelial collagen, enabling platelet tethering to the damaged vessel wall [28]. Upon platelet activation, CD42b is cleaved by metalloproteinases [29]. CD32a (FcγRIIa), the only Fc receptor expressed on platelets, is activated by immunoglobulin (Ig) G-containing immune complexes, which drive CD32a cross-linking and downstream platelet activation [30,31]. CD36 has a pathogenic role in platelets, as CD36-mediated platelet activation is driven by oxidized low-density lipoprotein [32]. The exact functional roles of CD147 and CXCR4 in platelets are unknown, but they are implicated in multiple proinflammatory conditions. CD147 has been implicated in SARS-CoV-2 [33] and HIV infection [34], and its main ligand, cyclophilin A, is released from various immune cells, including activated platelets [35]. The only known ligand of CXCR4 is the chemokine CXCL12, also known as stromal cell-derived factor 1. The latter is constitutively produced by stromal cells in a broad range of tissues, where interactions with CXCR4 drive cell survival and proliferation, chemotaxis, and gene transcription in various processes, including embryogenesis, tissue damage repair, and angiogenesis [36]. These inflammation-associated platelet surface receptors are elevated in various immune-mediated inflammatory diseases, where CD32a is elevated in heparin-induced thrombocytopenia [37], CD36 in inflammatory bowel disease [38], CD147 in rheumatoid arthritis [39], and CXCR4 in immune thrombocytopenia [40].

We assessed changes in platelet surface markers in response to different platelet agonists and inhibitors. We applied Full Annotation Using Shape-constrained Trees (FAUST; Tercen), an unsupervised machine learning algorithm that discovers and annotates statistically relevant cellular subpopulations within high-dimensional flow cytometry data on a per-sample basis [41], allowing us to define novel platelet subpopulations without concatenating datasets and the bias inherent to self-annotation of subpopulations. WB was stimulated with adenosine diphosphate (ADP), thrombin receptor-activating peptide-6 (TRAP-6), or convulxin (CVX). These biologically relevant agonists were selected for their ability to activate distinct platelet signaling pathways. By applying FAUST to this dataset, we identified 8 novel platelet subpopulations defined by differential levels of PAC1, CD62P, and CD63. To determine the effect of platelet inhibitors on platelet surface markers and platelet subpopulations, WB was pretreated with either prostacyclin (PGI_2_), dasatinib, vorapaxar, or ticagrelor prior to stimulation with ADP, TRAP-6, or CVX. These inhibitors were chosen specifically because they target ADP-, TRAP-6-, and/or CVX-mediated platelet activation. Using this approach, we show that platelet subpopulations are dynamic and can be remodeled in response to different agonists and inhibitors.

## 2 Methods

### 2.1 Reagents

Phosphate-buffered saline and ADP were from Millipore Sigma. Dasatinib, ticagrelor, vorapaxar, PGI_2_, and CVX were from Cayman Chemical. Dimethyl sulfoxide was from ChemCruz, 4% paraformaldehyde from Electron Microscopy Sciences, TRAP-6 from AnaSpec, and 100% ethanol from Honeywell Research Chemicals. Anti-mouse Ig κ/negative control compensation beads and PE mouse anti-human FcγRIIa (CD32a) were from BD Biosciences. All other antibodies were from BioLegend. Antibody clones and lot numbers are indicated in Supplementary Table S1.

### 2.2 Venipuncture

WB was drawn from healthy adult volunteers with informed consent and confirmed that they had not taken any medication that could affect platelet function (including aspirin and oral glucocorticoids) within 14 days prior to donation. Ethical approval was granted by the University Research Ethics Committee (MREC19-006), and the study was conducted in accordance with the Declaration of Helsinki. WB was collected into 2 mL vacutainers containing sodium citrate (3.2% trisodium citrate, 109 mM; Greiner Bio-One) 1:9 (v/v) [42]. The initial 2 mL of drawn blood was discarded to minimize tissue thromboplastin contamination and spontaneous platelet activation.

### 2.3 Flow cytometry

Platelet surface markers were analyzed by conventional flow cytometry. Antibodies (at titrated concentrations) and agonists (if present) were diluted in modified Tyrode’s buffer, creating the assay buffer. Freshly obtained WB [42] was diluted 1:9 in assay buffer, incubated for 20 minutes in the dark at room temperature, and then fixed with 1% paraformaldehyde/phosphate-buffered saline (1:10, v/v). When inhibitors were used, WB was pretreated with the inhibitor or its corresponding vehicle control for 15 minutes (dasatinib, ticagrelor, and vorapaxar) or 2 minutes (PGI_2_), and then incubated in assay buffer for 20 minutes at room temperature prior to fixation. Samples were analyzed using a Beckman Coulter CytoFLEX S flow cytometer equipped with 4 lasers (405 nm, 488 nm, 561 nm, and 640nm) and 8 filters (450/45 bandpass (BP), 525/40 BP, 585/42 BP, 610/20 BP, 660/20 BP, 690/50 BP, 712/25 BP, and 780/60 BP). Automatic compensation was performed using anti-mouse Ig κ/negative control compensation beads and CytExpert software (v2.5, Beckman Coulter). Data were acquired using CytExpert (v2.5), starting with manual gating of platelets as previously described [42]. In brief, platelets were isolated from WB/debris based on their distinct forward scatter vs side scatter (SSC) characteristics. Cells within this gate were confirmed as platelets by positivity for CD42b (CD42b^+^), and 10,000 CD42b^+^ events were recorded. Within the CD42b^+^ gate, doublets were excluded by removing events that showed an increased SSC area compared with SSC height. Median fluorescence intensity (MFI) for all 9 platelet surface markers was then determined from the remaining events.

### 2.4 FAUST analysis

**Supplementary Figure S1:**
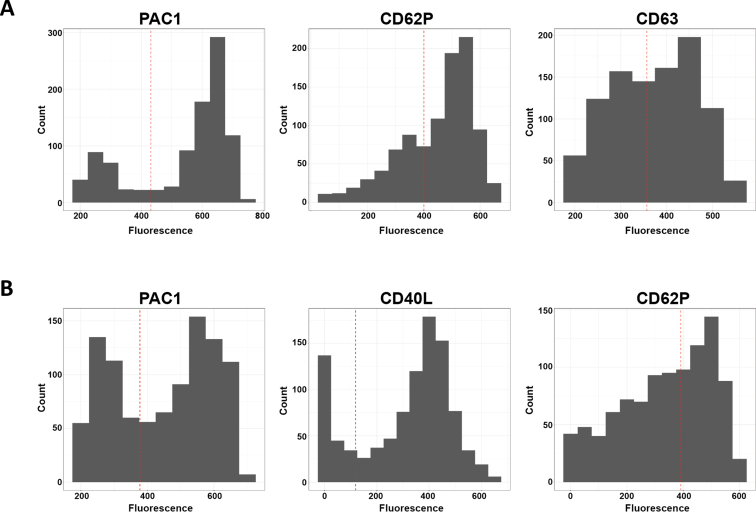


For subpopulation identification, FAUST (v.0.1.4; Tercen) was applied to WB flow cytometric data to capture measurements of CD62P, PAC1, CD40L, CD63, CD42b, CD147, CXCR4, CD32a, and CD36 when WB was stimulated with agonists in the presence and absence of inhibitors. FAUST is an unsupervised machine learning algorithm that discovers and annotates statistically relevant cellular subpopulations within high-dimensional flow cytometry data on a per-sample basis [41], allowing the user to define novel platelet subpopulations without concatenating datasets and without the bias inherent in self-annotation of subpopulations. FAUST requires multimodal expression of at least 1 marker within a dataset and assumes that markers showing multimodality between samples are informative and important for subpopulation annotation. FAUST performs automated and unsupervised thresholding of these markers to define negative and positive receptor expression, where anything below the threshold is defined as negative expression, and anything above the threshold is defined as positive expression (Supplementary Figure S1).

Data acquisition, manual gating, and compensation were performed using CytExpert. Data from single platelets were exported into FlowJo for biexponential transformation as previously described [43]. To determine multimodality of all 9 platelet surface markers, fluorescence intensities were converted into channel values in FlowJo. Data were then exported to the Tercen platform for FAUST analysis. Down-sampling of data was not conducted, as all platelets from all volunteers were analyzed (> 34,000 platelets per sample from 4 independent volunteers). To visualize the clustered high-dimensional data, FAUST subpopulations underwent dimension reduction and were embedded in uniform manifold approximation and projections, which were then exported to FlowJo for the determination of individual frequencies and marker fluorescence levels.

### 2.5 Statistical analysis

Statistical analyses were performed using GraphPad Prism (v10). Data are expressed as mean ± SEM and analyzed using one-way analysis of variance (anova) followed by multiple comparison post hoc tests (specific tests are described in figure legends), unless stated otherwise. Statistical significance was assumed at *P* < .05. All statistically significant comparisons mentioned within the results section are shown in Supplementary Table S2.

## 3 Results

### 3.1 Multiparameter flow cytometry identifies changes in platelet surface markers in response to platelet activation

**Figure 1:**
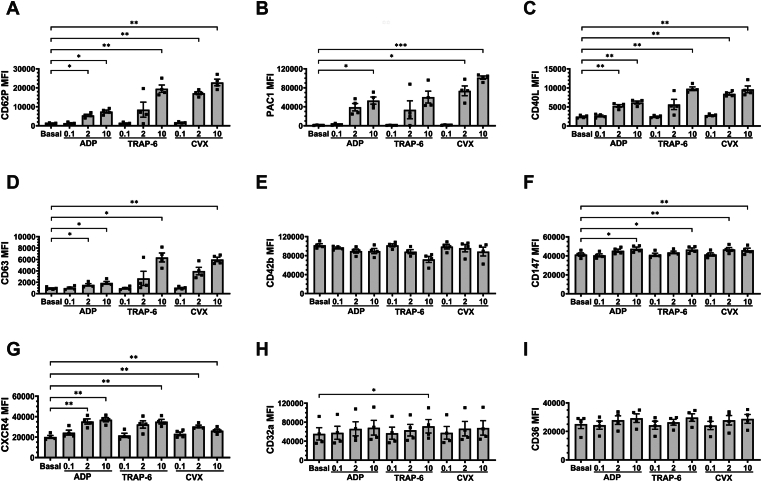
Dose-dependent effects of adenosine diphosphate (ADP)-, thrombin receptor-activating peptide-6 (TRAP-6)-, and convulxin (CVX)-mediated platelet activation on platelet surface markers. Whole blood was unstimulated (basal) or stimulated with varying concentrations of ADP (0.1-10 μM), TRAP-6 (0.1-10 μM), or CVX (0.1-10 ng/mL) for 20 minutes prior to fixation. Fixed samples were then analyzed by flow cytometry, where the median fluorescence intensity (MFI) of (A) CD62P, (B) PAC1, (C) CD40L, (D) CD63, (E) CD42b, (F) CD147, (G) CXCR4, (H) CD32a, and (I) CD36 was quantified. Data are expressed as mean ± SEM and were analyzed by repeated measures one-way anova followed by Dunnett’s multiple comparison test (*n* = 4). ∗*P* ≤ .05, ∗∗*P* ≤ .01, ∗∗∗*P* ≤ .001.

First, we examined the concentration-dependent effects of agonists on platelet surface markers when WB was treated with ADP (0.1-10 μM), TRAP-6 (0.1-10 μM), or CVX (0.1-10 ng/mL; Figure 1). High concentrations of ADP (10 μM), TRAP-6 (10 μM), and CVX (10 ng/mL) drove significant increases in CD62P, CD40L, CD63, CD147, and CXCR4 MFI. PAC1 MFI was significantly increased by high concentrations of ADP and CVX, and CD32a MFI was significantly increased by high concentrations of TRAP-6. Intermediate concentrations of ADP (2 μM) and CVX (2 ng/mL) drove significant increases in CD62P, CD40L, and CXCR4 MFI. PAC1 and CD147 MFI were significantly increased by intermediate concentrations of CVX, and CD63 MFI was significantly increased by intermediate concentrations of ADP.

Intermediate concentrations of TRAP-6 (2 μM) did not induce significant increases in any platelet surface marker. This was true for low concentrations of ADP (0.1 μM), TRAP-6 (0.1 μM), and CVX (0.1 ng/mL). Interestingly, we observed interdonor variations in platelet surface marker levels in response to intermediate concentrations of TRAP-6 and CVX. This was particularly noticeable with CD62P, PAC1, and CD63 MFI, suggesting that healthy adults demonstrate variations in sensitivity to intermediate concentrations of these agonists. Interestingly, we found that increases in MFI were more profound for the 4 activation-induced markers (CD62P, PAC1, CD40L, and CD63) compared with constitutively expressed markers (CD42b, CD147, CXCR4, CD32a, and CD36). Taken together, these data show that our panel can detect changes in platelet surface markers when WB is stimulated with varying doses of ADP, TRAP-6, or CVX.

### 3.2 Multiparameter flow cytometry identifies distinct platelet subpopulations in response to platelet activation

**Figure 2:**
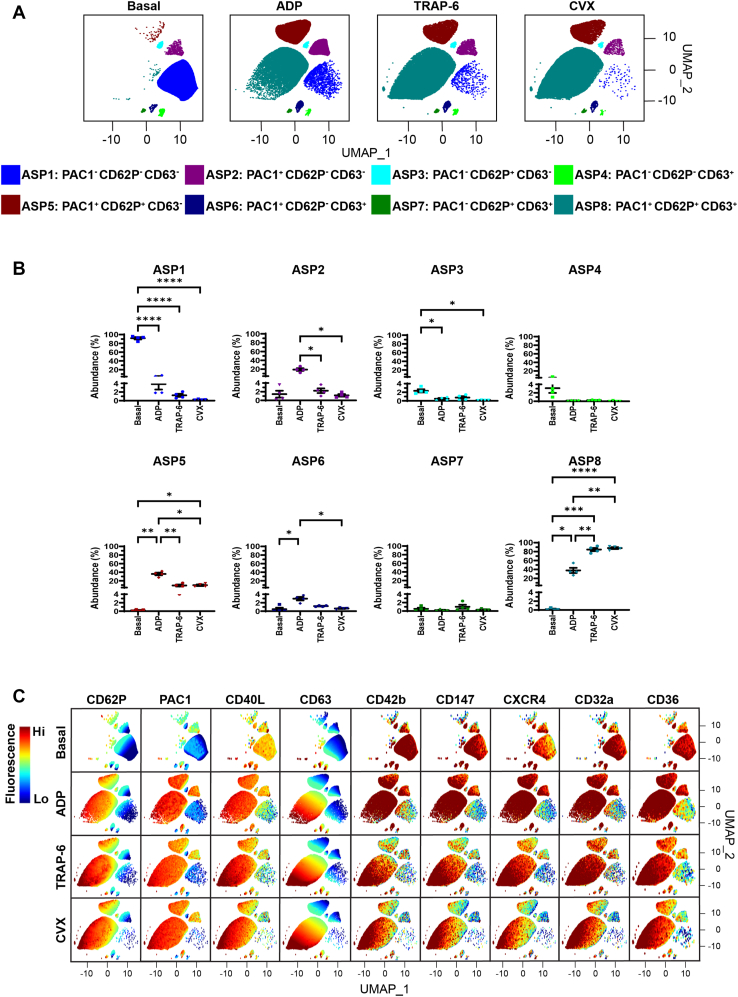
The effect of adenosine diphosphate (ADP)-, thrombin receptor-activating peptide-6 (TRAP-6)-, and convulxin (CVX)-mediated platelet activation on platelet subpopulations. Whole blood was unstimulated (basal) or stimulated with ADP (10 μM), TRAP-6 (10 μM), or CVX (10 ng/mL) for 20 minutes prior to fixation. Fixed samples were then analyzed by flow cytometry, where the fluorescence intensity of CD62P, PAC1, CD40L, CD63, CD42b, CD147, CXCR4, CD32a, and CD36 was quantified. Platelet subpopulation analysis was then conducted by Full Annotation Using Shape-constrained Trees, which identified 8 unique agonist-associated subpopulations (ASPs) defined and annotated by PAC1, CD62P, and CD63 fluorescence. (A) Platelet subpopulations from 4 donors are visualized in uniform manifold approximation and projection (UMAP) graphs. (B) Plots showing the changes in ASP1 to 8 abundance between conditions. Data are expressed as mean ± SEM and were analyzed by repeated measures one-way anova followed by Tukey’s multiple comparison test (*n* = 4). ∗*P* ≤ .05, ∗∗*P* ≤ .01, ∗∗∗*P* ≤ .001, ∗∗∗∗*P* ≤ .0001. (C) UMAPs showing the fluorescence of CD62P, PAC1, CD40L, CD63, CD42b, CD147, CXCR4, CD32a, and CD36 within ASP1 to 8. The color scale represents the z-score derived from platelet surface marker fluorescence (explained in the Methods). Hi, high; Lo, low.

Next, we characterized the platelet subpopulations present at baseline and after stimulation with ADP (10 μM), TRAP-6 (10 μM), or CVX (10 ng/mL). FAUST identified 8 distinct platelet subpopulations based on the differential expression of PAC1, CD62P, and CD63 (Figure 2A). The subpopulations were labeled agonist-associated subpopulations (ASPs) 1 to 8, and they were defined as resting platelets (ASP1; PAC1^-^CD62P^-^CD63^-^), platelets with activated α_IIb_β_3_ (ASP2; PAC1^+^CD62P^-^CD63^-^), platelets with α-granule secretion (ASP3; PAC1^-^CD62P^+^CD63^-^), platelets with dense granule/lysosome secretion (ASP4; PAC1^-^CD62P^-^CD63^+^), platelets with activated α_IIb_β_3_ and α-granule secretion (ASP5; PAC1^+^CD62P^+^CD63^-^), platelets with activated α_IIb_β_3_ and dense granule/lysosome secretion (ASP6; PAC1^+^CD62P^-^CD63^+^), platelets with full degranulation (ASP7; PAC1^-^CD62P^+^CD63^+^), and fully activated platelets with activated α_IIb_β_3_ and full degranulation (ASP8; PAC1^+^CD62P^+^CD63^+^). The automated thresholds that were determined by FAUST for PAC1, CD62P, and CD63 are shown in Supplementary Figure S1A.

Under basal conditions, the most abundant platelet subpopulation was ASP1 (91.79% ± 2.65%), followed by ASP4 (3.11% ± 1.13%), ASP3 (2.34% ± 0.37%), and ASP2 (1.45% ± 0.79%; Figure 2B). These data are consistent with most of the total platelet population being quiescent *in vivo*. Stimulation of platelets by all 3 agonists induced a remodeling of these subpopulations, leading to a significant decrease in ASP1 and a significant increase in ASP8 (Figure 2B). TRAP-6 and CVX were similar in their ability to stimulate maximal platelet activation, with high levels of ASP8 emerging; no significant differences occurred between the 2 agonists. TRAP-6 and CVX induced a greater increase in ASP8 than ADP. Moreover, ADP significantly increased the abundance of subpopulations that were not fully activated and thus did not express all 3 activation markers, such as ASP2 and ASP5, in comparison with TRAP-6 and CVX (Figure 2B). This suggests that ADP is weaker at stimulating simultaneous secretion of α-granules, dense granules, and lysosomes.

**Figure 3:**
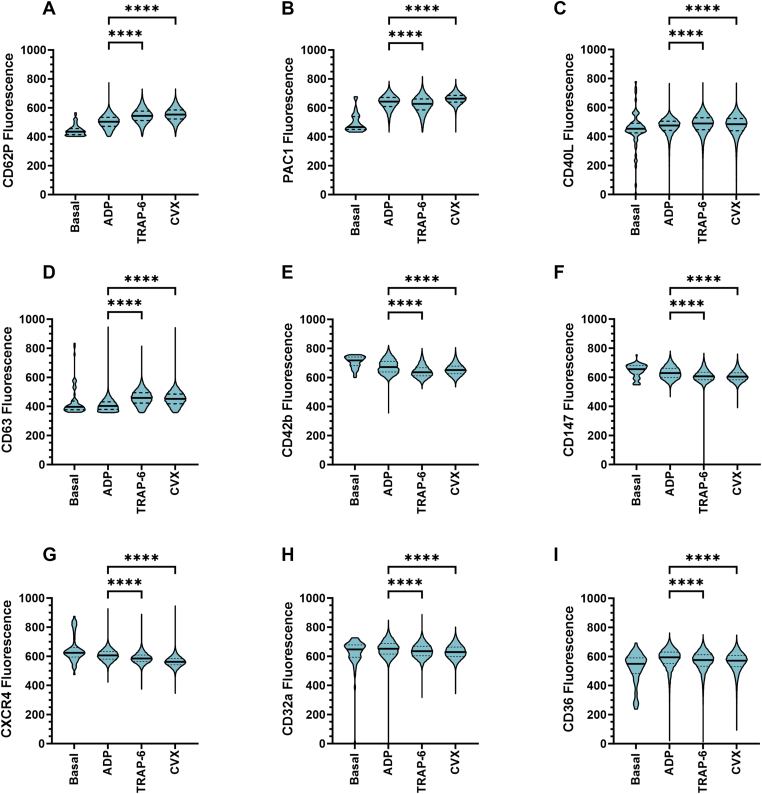
The effect of adenosine diphosphate (ADP)-, thrombin receptor-activating peptide-6 (TRAP-6)-, and convulxin (CVX)-mediated platelet activation on platelet surface markers within agonist-associated subpopulations (ASPs) 1 to 8. Whole blood was unstimulated (basal) or stimulated with ADP (10 μM), TRAP-6 (10 μM), or CVX (10 ng/mL) for 20 minutes prior to fixation. Fixed samples from 4 independent donors were then analyzed by flow cytometry, where the median fluorescence intensity of CD62P, PAC1, CD40L, CD63, CD42b, CD147, CXCR4, CD32a, and CD36 was quantified. Platelet subpopulation analysis was conducted using Full Annotation Using Shape-constrained Trees, which identified 8 unique ASPs defined and annotated by PAC1, CD62P, and CD63 fluorescence. Plots showing the changes in (A) CD62P, (B) PAC1, (C) CD40L, (D) CD63, (E) CD42b, (F) CD147, (G) CXCR4, (H) CD32a, and (I) CD36 fluorescence within ASP8 (PAC1^+^CD62P^+^CD63^+^) between conditions. Data are shown as violin plots, where the solid black line represents the median and the dotted lines represent the 25th and 75th percentiles. Data were analyzed using the Kruskal–Wallis test followed by Dunn’s multiple comparison test. ∗*P* ≤ .05, ∗∗*P* ≤ .01, ∗∗∗*P* ≤ .001, ∗∗∗∗*P* ≤ .0001.

By examining the fluorescence of all platelet surface markers within each platelet subpopulation (Figure 2C), we found that the fully activated subpopulation (ASP8) was also enriched in CD40L, another α-granule protein that is secreted upon platelet activation [17]. Interestingly, within this subpopulation, there was a gradation of CD63 fluorescence in response to all 3 agonists, with some platelets showing high expression and others showing low expression, suggesting that there may be additional rare subdivisions within this subpopulation that have differential CD63 expression patterns. Moreover, we also observed differences in ASP8 that were potentially agonist-dependent. For example, TRAP-6 and/or CVX induced greater changes in CD62P, PAC1, CD40L, CD63, and CD42b fluorescence than ADP, but ADP induced greater changes in CD147, CXCR4, CD32a, and CD36 fluorescence (Figure 3). Taken together, these data show that, in response to ADP, TRAP-6, or CVX, unique platelet subpopulations are defined by differential expression of PAC1, CD62P, and CD63, but subpopulation and platelet surface marker abundance within subpopulations is agonist-dependent.

### 3.3 Multiparameter flow cytometry identifies differences in agonist sensitivity to platelet inhibitors

To assess the robustness of our panel across different assays, we examined the effects of platelet inhibitors on platelet surface markers. WB was pretreated with PGI_2_, dasatinib, vorapaxar, ticagrelor, or their respective vehicle controls, followed by stimulation with 10 μM ADP, 10 μM TRAP-6, or 2 ng/mL CVX. The lower CVX concentration was used in place of 10 ng/mL, as the higher dose resulted in insufficient inhibition (data not shown). We found no significant difference in platelet surface marker MFI when WB was stimulated with agonists in the absence vs the presence of vehicle controls (Supplementary Figure S2). Therefore, all comparisons in this section focus on agonist stimulation in the absence vs the presence of an inhibitor.

**Figure 4:**
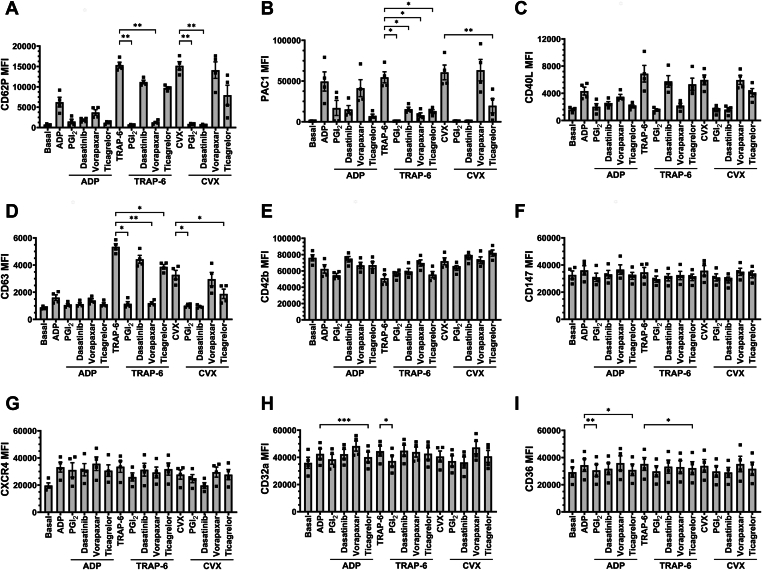
The effect of platelet inhibitors on adenosine diphosphate (ADP)-, thrombin receptor-activating peptide-6 (TRAP-6)-, and convulxin (CVX)-mediated platelet activation and platelet surface markers. Whole blood was unstimulated (basal) or stimulated with ADP (10 μM), TRAP-6 (10 μM), or CVX (2 ng/mL) for 20 minutes prior to fixation. When inhibitors were used, whole blood was pretreated with 5 nM prostacyclin (PGI_2_) for 2 minutes, or with 500 nM dasatinib, 1 μM vorapaxar, or 1 μM ticagrelor for 15 minutes prior to agonist stimulation. Fixed samples were then analyzed by flow cytometry, where the median fluorescence intensity (MFI) of (A) CD62P, (B) PAC1, (C) CD40L, (D) CD63, (E) CD42b, (F) CD147, (G) CXCR4, (H) CD32a, and (I) CD36 was quantified. Data are expressed as mean ± SEM and were analyzed by repeated measures one-way anova followed by Šídák’s multiple comparison test (*n* = 4). ∗*P* ≤ .05, ∗∗*P* ≤ .01, ∗∗∗*P* ≤ .001, ∗∗∗∗*P* ≤ .0001.

We found that PGI_2_ and ticagrelor targeted all 3 activation pathways, whereas dasatinib affected TRAP-6- and CVX-mediated platelet activation, and vorapaxar affected TRAP-6–mediated platelet activation. Despite this, the inhibitors had differential effects on platelet surface markers (Figure 4). PGI_2_ significantly reduced CD62P and CD63 MFI in response to TRAP-6 and CVX, PAC1 and CD63 MFI in response to TRAP-6, and CD36 MFI in response to ADP. Dasatinib significantly reduced CD62P MFI in response to CVX and PAC1 MFI in response to TRAP-6. Vorapaxar significantly reduced CD62P, PAC1, and CD63 MFI in response to TRAP-6. Ticagrelor significantly reduced PAC1 and CD63 MFI in response to TRAP-6 and CVX, CD36 MFI in response to ADP and TRAP-6, and CD32a MFI in response to ADP. Taken together, these data show that our panel can detect changes in platelet surface markers when WB is treated with different platelet inhibitors prior to agonist stimulation.

### 3.4 Multiparameter flow cytometry identifies how platelet inhibitors influence the formation of platelet subpopulations in response to platelet activation

**Figure 5:**
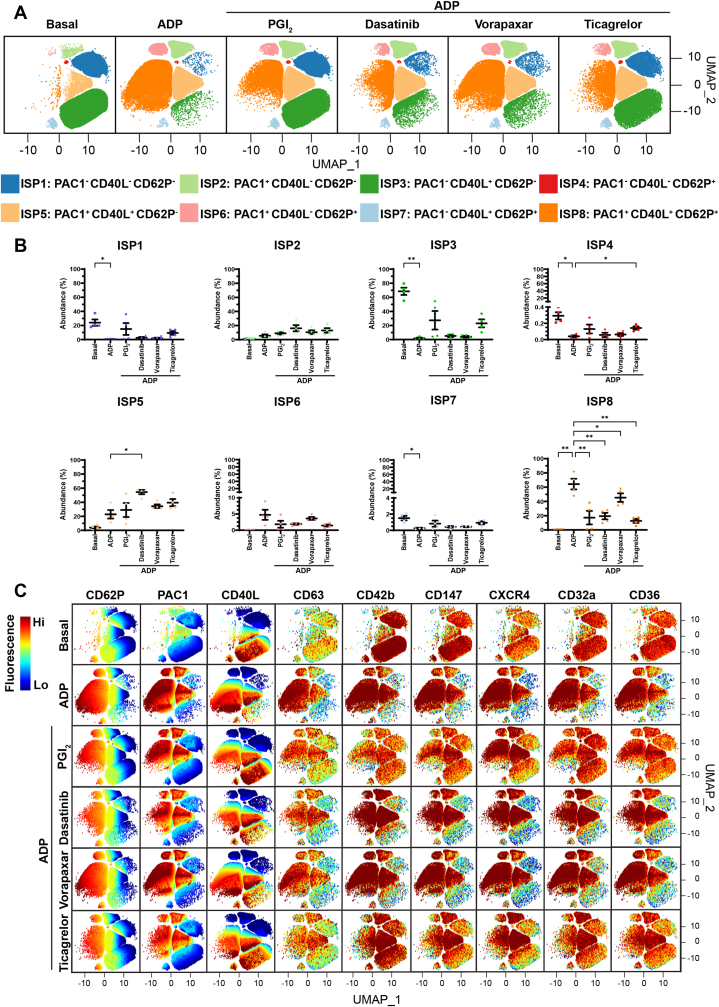
The effect of platelet inhibitors on adenosine diphosphate (ADP)-mediated platelet activation and platelet subpopulations. Whole blood was unstimulated (basal) or stimulated with ADP (10 μM) for 20 minutes prior to fixation. When inhibitors were used, whole blood was pretreated with 5 nM prostacyclin (PGI_2_) for 2 minutes, or with 500 nM dasatinib, 1 μM vorapaxar, or 1 μM ticagrelor for 15 minutes prior to agonist stimulation. Fixed samples were then analyzed by flow cytometry, where the fluorescence intensity of CD62P, PAC1, CD40L, CD63, CD42b, CD147, CXCR4, CD32a, and CD36 was quantified. Platelet subpopulation analysis was then conducted using Full Annotation Using Shape-constrained Trees, which identified 8 unique inhibitor-associated subpopulations (ISPs) defined and annotated by PAC1, CD40L, and CD62P fluorescence. (A) Platelet subpopulations from 4 donors are visualized in uniform manifold approximation and projection (UMAP) graphs. (B) Plots showing the changes in ISP1 to 8 abundance between conditions. Data are expressed as mean ± SEM and were analyzed by repeated measures one-way anova followed by Dunnett’s multiple comparison test (*n* = 4). ∗*P* ≤ .05, ∗∗*P* ≤ .01. (C) UMAPs showing the fluorescence of CD62P, PAC1, CD40L, CD63, CD42b, CD147, CXCR4, CD32a, and CD36 within ISP1 to 8. The color scale represents the z-score derived from platelet surface marker fluorescence (explained in the Methods). Hi, high; Lo, low.

**Figure 6:**
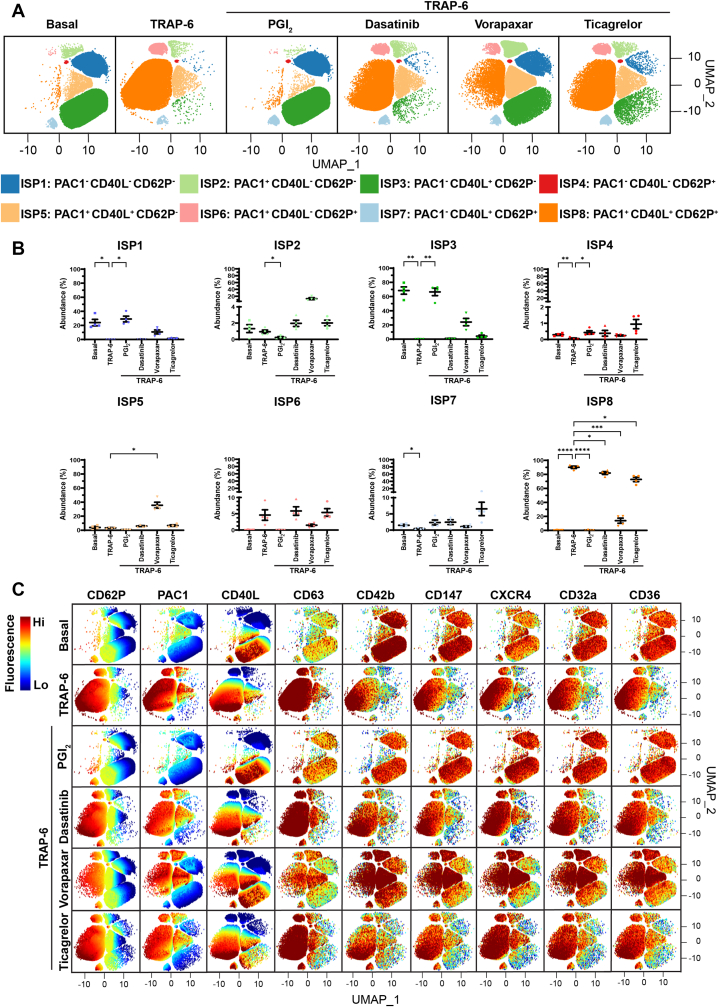
The effect of platelet inhibitors on thrombin receptor-activating peptide-6 (TRAP-6)-mediated platelet activation and platelet subpopulations. Whole blood was unstimulated (basal) or stimulated with TRAP-6 (10 μM) for 20 minutes prior to fixation. When inhibitors were used, whole blood was pretreated with 5 nM prostacyclin (PGI_2_) for 2 minutes, or with 500 nM dasatinib, 1 μM vorapaxar, or 1 μM ticagrelor for 15 minutes prior to agonist stimulation. Fixed samples were then analyzed by flow cytometry, where the fluorescence intensity of CD62P, PAC1, CD40L, CD63, CD42b, CD147, CXCR4, CD32a, and CD36 was quantified. Platelet subpopulation analysis was then conducted using Full Annotation Using Shape-constrained Trees, which identified 8 unique inhibitor-associated subpopulations (ISPs) defined and annotated by PAC1, CD40L, and CD62P fluorescence. (A) Platelet subpopulations from 4 donors are visualized in uniform manifold approximation and projection (UMAP) graphs. (B) Plots showing the changes in ISP1 to 8 abundance between conditions. Data are expressed as mean ± SEM and were analyzed by repeated measures one-way anova followed by Dunnett’s multiple comparison test (*n* = 4). ∗*P* ≤ .05, ∗∗*P* ≤ .01, ∗∗∗*P* ≤ .001, ∗∗∗∗*P* ≤ .0001. (C) UMAPs showing the fluorescence of CD62P, PAC1, CD40L, CD63, CD42b, CD147, CXCR4, CD32a, and CD36 within ISP1 to 8. The color scale represents the z-score derived from platelet surface marker fluorescence (explained in the Methods). Hi, high; Lo, low.

**Figure 7:**
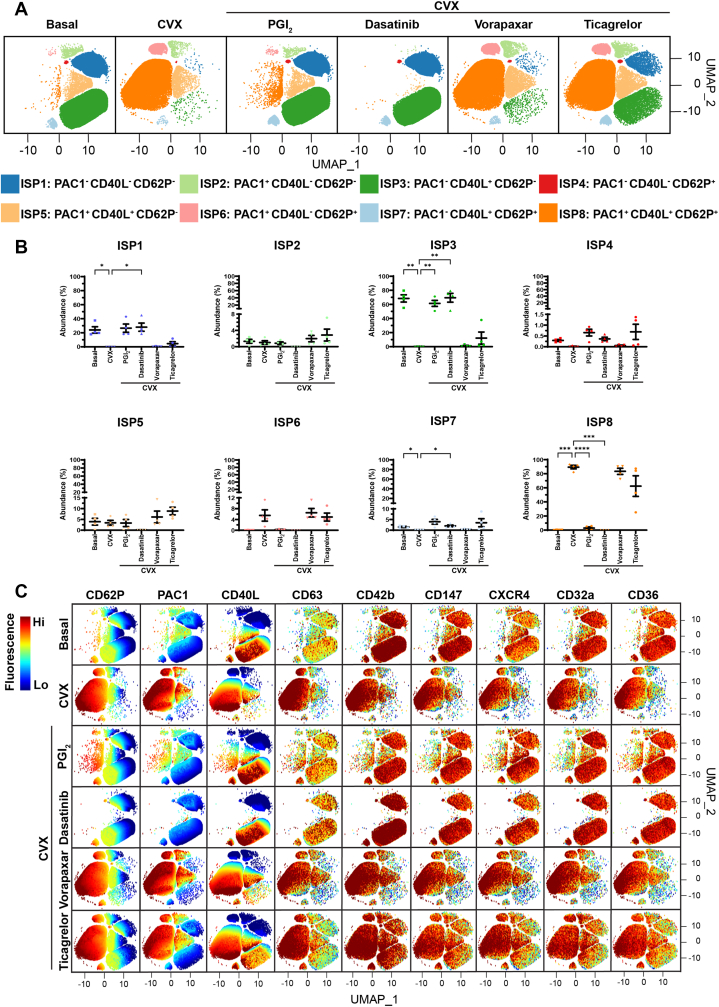
The effect of platelet inhibitors on convulxin (CVX)-mediated platelet activation and platelet subpopulations. Whole blood was unstimulated (basal) or stimulated with CVX (2 ng/mL) for 20 minutes prior to fixation. When inhibitors were used, whole blood was pretreated with 5 nM prostacyclin (PGI_2_) for 2 minutes, or with 500 nM dasatinib, 1 μM vorapaxar, or 1 μM ticagrelor for 15 minutes prior to agonist stimulation. Fixed samples were then analyzed by flow cytometry, where the fluorescence intensity of CD62P, PAC1, CD40L, CD63, CD42b, CD147, CXCR4, CD32a, and CD36 was quantified. Platelet subpopulation analysis was then conducted using Full Annotation Using Shape-constrained Trees, which identified 8 unique inhibitor-associated subpopulations (ISPs) defined and annotated by PAC1, CD40L, and CD62P fluorescence. (A) Platelet subpopulations from 4 donors are visualized in uniform manifold approximation and projection (UMAP) graphs. (B) Plots showing the changes in the abundance of ISP1 to 8 between conditions. Data are expressed as mean ± SEM and were analyzed by repeated measures one-way anova followed by Dunnett’s multiple comparison test (*n* = 4). ∗*P* ≤ .05, ∗∗*P* ≤ .01, ∗∗∗*P* ≤ .001, ∗∗∗∗*P* ≤ .0001. (C) UMAPs showing the fluorescence of CD62P, PAC1, CD40L, CD63, CD42b, CD147, CXCR4, CD32a, and CD36 within ISP1 to 8. The color scale represents the z-score derived from platelet surface marker fluorescence (explained in the Methods). Hi, high; Lo, low.

Next, we characterized the platelet subpopulations present when WB was treated with PGI_2_, dasatinib, vorapaxar, or ticagrelor prior to agonist stimulation with ADP (10 μM), TRAP-6 (10 μM), or CVX (2 ng/mL). FAUST identified 8 distinct platelet subpopulations based on differential expression of PAC1, CD40L, and CD62P (Figure 5, Figure 6, Figure 7), which were labeled inhibitor-associated subpopulations (ISPs) 1 to 8. This was because they differed from the subpopulations that were present when WB was stimulated with agonists in the absence of inhibitors. ISP1 to 8 were defined as resting platelets (ISP1; PAC1^-^CD40L^-^CD62P^-^), platelets with activated α_IIb_β_3_ (ISP2; PAC1^+^CD40L^-^CD62P^-^), platelets with α-granule secretion but only CD40L expression (ISP3; PAC1^-^CD40L^+^CD62P^-^), platelets with α-granule secretion but only CD62P expression (ISP4; PAC1^-^CD40L^-^CD62P^+^), platelets with activated α_IIb_β_3_ and α-granule secretion but only CD40L expression (ISP5; PAC1^+^CD40L^+^CD62P^-^), platelets with activated α_IIb_β_3_ and α-granule secretion but only CD62P expression (ISP6; PAC1^+^CD40L^-^CD62P^+^), platelets with α-granule secretion and both CD62P and CD40L expression (ISP7; PAC1^-^CD40L^+^CD62P^+^), and platelets with activated α_IIb_β_3_ and α-granule secretion with both CD40L and CD62P expression (ISP8; PAC1^+^CD40L^+^CD62P^+^). The automated thresholds that were determined by FAUST for PAC1, CD40L, and CD62P are shown in Supplementary Figure S1B.

Under basal conditions, the most prominent subpopulation was ISP3 (68.49% ± 5.13%), followed by ISP1 (24.01% ± 4.53%) and ISP5 (3.94% ± 1.55%; Figure 5, Figure 6, Figure 7). Platelets within ISP3 showed variation in CD40L expression, with some platelets showing dim fluorescence and others showing high fluorescence (Figures 5C, 6C, and 7C). Platelets in this subpopulation appeared to show a continuum of high CD40L expression, suggesting that within this subpopulation, there may be additional subpopulations with unique CD40L expression patterns and differing levels of platelet activation. Stimulation with all 3 agonists induced a significant increase in ISP8 abundance, indicative of full platelet activation, as platelets within this subpopulation also expressed CD63 (Figures 5C, 6C, and 7C), consistent with dense granule/lysosome secretion alongside α-granule secretion and α_IIb_β_3_ activation. All inhibitors were associated with significant decreases in the abundance of ISP8 in response to ADP and TRAP-6 (Figures 5B and 6B), whereas PGI_2_ and dasatinib were associated with significant decreases in the abundance of ISP8 in response to CVX (Figure 7B). Preincubation with PGI_2_ ablated the changes in ISP1, ISP3, and ISP8 generated by TRAP-6 and CVX (Figures 6B and 7B), with abundances comparable to those in basal conditions. This was also seen when WB was pretreated with dasatinib followed by stimulation with CVX (Figure 7B). These data demonstrate that, at the concentrations used in these experiments, PGI_2_ completely inhibits TRAP-6- and CVX-mediated platelet activation, and dasatinib completely inhibits CVX-mediated platelet activation.

Ticagrelor was associated with a significant increase in ISP4 abundance in response to ADP (Figure 5B), suggesting that in some platelets, ticagrelor influences α_IIb_β_3_ activation and CD40L expression but not CD62P expression. Critically, pretreatment of WB with dasatinib or vorapaxar prior to stimulation with ADP or TRAP-6 (Figures 5B and 6B), respectively, was associated with significant increases in the abundance of ISP5. Moreover, dasatinib and ticagrelor had weak effects on TRAP-6–mediated platelet activation (Figure 6), suggesting that TRAP-6 signals more through phospholipase C_β_ than through Src-family kinases, and is not reliant on secondary activation by ADP. Taken together, these experiments demonstrate that the platelet inhibitors have differential effects on platelet subpopulations when platelets are stimulated with different agonists.

## 4 Discussion

Emerging data indicate that platelets may play functionally diverse roles, with implications for their assessment and pharmacological targeting in a range of disorders. Furthermore, platelets show high levels of functional plasticity, potentially tailoring their response to different agonists under specific conditions. With this in mind, we designed and tested a novel multiparameter flow cytometry panel that analyzed changes in 9 platelet surface markers. This was coupled with a new interpretable machine learning method, called FAUST, to determine whether distinct subpopulations of platelets were associated with specific agonists and how this was affected by currently used pharmacological agents that inhibit platelet activation. The panel was designed to simultaneously measure a combination of platelet surface activation-induced (CD62P, PAC1, CD40L, and CD63) and constitutive (CD42b, CD147, CXCR4, CD32a, and CD36) markers. In the initial experiments, WB was stimulated with ADP, TRAP-6, or CVX. In these experiments, FAUST analysis found 8 platelet subpopulations (ASP1-8), which were defined by differential levels of PAC1, CD62P, and CD63 (Figure 2). At rest, most platelets were quiescent (ASP1), but platelet activation led to subpopulation remodeling, where platelets began to express activation markers, resulting in most platelets becoming PAC1^+^, CD62P^+^, and CD63^+^ (ASP8). These data imply that platelet subpopulations are dynamic and change in response to stimulation by agonists. ADP generated significantly fewer fully activated platelets (ASP8) and significantly more platelets that did not show full platelet degranulation and thus did not express all 3 activation markers, such as ASP2 and ASP5, showing that ADP is weaker at driving full platelet activation in comparison with TRAP-6 and CVX.

While platelets play key roles in hemostasis and innate immunity, maladaptive function is associated with a plethora of diseases, including type 2 diabetes [44], cardiovascular disease [45], rheumatoid arthritis [46], and giant cell arteritis [47]. Therefore, antiplatelet medication is a critical element for a multitude of clinical situations. Thus, it is important to understand whether antiplatelet medications affect platelets uniformly or if their effects are targeted to specific subpopulations. WB was pretreated with PGI_2_, dasatinib, vorapaxar, or ticagrelor prior to ADP, TRAP-6, or CVX stimulation. We observed variations in platelet surface marker MFI in response to some inhibitors (Figure 4), which may be due to variations in donor sensitivity to agonists (Figure 1) because of platelet age and varying receptor expression [3]. These factors may affect inhibitor efficacy, particularly for those inhibitors that interact directly with surface receptors, such as vorapaxar and ticagrelor. In these experiments, FAUST analysis discovered the presence of 8 platelet subpopulations (ISP1-8), although annotations of these subpopulations were determined by differential levels of PAC1, CD40L, and CD62P. Thus, a key finding is that under conditions of platelet inhibition, FAUST selects CD40L expression for annotation. We found that there were subpopulations present that demonstrated α-granule secretion but did not coexpress CD62P and CD40L, such as ISP3, ISP4, ISP5, and ISP6. Platelets are thought to secrete both CD40L and CD62P from their α-granules [16,17], but our data suggest that α-granule cargo release is not uniform, a finding previously reported by Hermann et al. [48], who found that stimulation with low concentrations of collagen induced CD40L but not CD62P expression. Differential release of other α-granule proteins has been documented, such as von Willebrand factor and fibrinogen [49], as well as endostatin and vascular endothelial growth factor [50]. Hermann et al. [48] also showed that CD62P is found within granules, whereas CD40L is detected within the cytoplasm and associates with cytoplasmic actin filaments and the membrane skeleton [48]. This may explain why CD62P and CD40L expression seemed to occur independently of each other and may also explain why pretreatment of WB with dasatinib or vorapaxar, prior to stimulation with ADP or TRAP-6, respectively, was associated with significant increases in the abundance of ISP5.

Another unexpected finding was that vorapaxar was associated with significant reductions in ISP8 abundance in response to ADP, as vorapaxar had no effect on the MFI of any platelet surface marker in response to ADP. This finding may be a consequence of thrombin generation contributing to ADP-mediated platelet activation, even though experiments were conducted in citrated WB. Jiang et al. [51] have previously shown that in platelet-rich plasma isolated from citrated WB, thrombin, acting through protease-activated receptor 1, drives the second wave of ADP-induced platelet aggregation and degranulation, thus contributing to ADP-mediated platelet activation, suggesting that thrombin generation is independent of calcium. These data suggest that maximal ADP-mediated platelet activation is reliant on PAR1 signaling.

Developed by Green et al. [41] in 2021, FAUST is a new machine learning algorithm that has been applied to high-dimensional cytometry data across different cell types [41,[52], [53], [54]] and offers 3 main advantages over commonly used algorithms, such as FlowSOM [55], t-distributed stochastic neighbor embedding (t-SNE) [56], and SPADE [57]. First, the use of unsupervised analysis in our study allowed FAUST to find platelet subpopulations within unlabeled data without any prior assumptions. This reduces bias, which can occur when using FlowSOM, as the algorithm requires the researcher to predetermine the number of clusters present within the dataset prior to analysis. When analyzing novel datasets, predetermining clusters is not possible and therefore requires the researcher to make strong assumptions about their data prior to analysis. Second, data concatenation was not required, as FAUST looks for trends and patterns within datasets on a sample-by-sample basis. This allows subpopulations unique to specific samples and/or participants to be identified for further analysis. With algorithms such as t-SNE, samples must be analyzed independently of each other, which can make comparisons between samples difficult, unless data sets are concatenated, which can result in rare sample-specific subpopulations being missed. Third, self-annotation of subpopulations was not required, as FAUST labels subpopulations, thereby increasing reproducibility by standardizing annotations and removing researcher bias. With FlowSOM, t-SNE, and SPADE analysis, researchers must self-annotate clusters, which can introduce variability as researchers may label clusters differently.

A limitation of automated thresholding in this study is that rarer subpopulations, particularly those not consistently present across all conditions or participants, may be overlooked. This may be the case in Figures 2 and Figure 5, Figure 6, Figure 7, where variations in CD63 and CD40L expression were apparent within certain subpopulations. Identifying such populations would be more feasible using FAUST within a programming environment such as R (R Project), where users can manually set thresholds using fluorescence minus one controls or define multiple thresholds to distinguish low, medium, and high expression levels of a given marker with a multimodal distribution. Another limitation of automated thresholding is that when datasets were analyzed separately, as in Figures 3 and Figure 5, Figure 6, Figure 7, the thresholds used to define marker positivity differed, as they were determined independently for each dataset. FAUST determines thresholds based on the distribution of signals within each dataset, and because the datasets differ in terms of participants, treatments, and experimental runs, variation in signal distributions (and thus threshold placement) is expected. For example, the ASP and ISP datasets were generated using different CVX concentrations, experimental goals, and donor populations. Consequently, the thresholds applied to markers that define the ASP1 to 8 and ISP1 to 8 subpopulations differ slightly. Merging them for a combined analysis without correcting for batch effects would likely introduce more noise than clarity. Due to this limitation, we do not compare the identified subpopulations across datasets. Researchers using this methodology should carefully plan their experimental design if cross-dataset comparisons are intended, and consider the need to address batch effects explicitly.

Characterizing platelet subpopulations present under physiological conditions is a crucial step toward understanding how platelets contribute to disease, as distinct subpopulations may have unique effector functions that drive specific pathological phenotypes. Applying this pipeline to analyze the effects of pathological ligands or phenotype platelets in disease contexts may reveal subpopulations annotated by different platelet surface markers. In this study, the primary drivers of platelet subpopulation annotation were the 4 activation-induced markers: PAC1, CD62P, CD63, and CD40L. This likely reflects the nature of the stimuli used (ADP, TRAP-6, and CVX), which are potent hemostatic agonists known to predominantly engage classical platelet activation pathways. Although not used for subpopulation annotation, CD147, CD32a, CXCR4, and CD36 respond dynamically to agonist stimulation. For example, we show that ADP induces greater increases in CD147, CD32a, CXCR4, and CD36 fluorescence compared with TRAP-6 or CVX within ASP8, suggesting that ADP may more strongly engage inflammation-related signaling pathways. These findings add novel insights to the literature on agonist-specific platelet responses. While these markers may not have played a defining role in subpopulation annotation in healthy donor samples, they may be more informative in disease states. This panel is currently being utilized to phenotype platelets in immune-mediated inflammatory disease, where platelets are exposed to proinflammatory stimuli such as oxidized low-density lipoprotein [[58], [59], [60]], cytokines [61,62], or immune complexes [[63], [64], [65]]. These pathogenic ligands can upregulate inflammation-associated markers and potentially define rare or disease-specific subpopulations.

In conclusion, this methodology can be used for high-throughput evaluation of the effects of pathological ligands, new pharmacological agents, and/or disease status on platelet surface receptor expression and platelet subpopulations.

## Figures and Tables

**Supplementary Figure S2:**