# Supplementary material for: Phenotyping of human platelets in response to platelet agonists and inhibitors using multiparameter flow cytometry and unbiased high-dimensional analysis
Source: Res Pract Thromb Haemost. 2025 Sep 23;9(7):103189. doi: 10.1016/j.rpth.2025.103189 (PMC12589989; doi:10.1016/j.rpth.2025.103189)
Supplement: Supplementary Tables S1-S2 [file mmc1.docx]

**Supplementary Material**

**
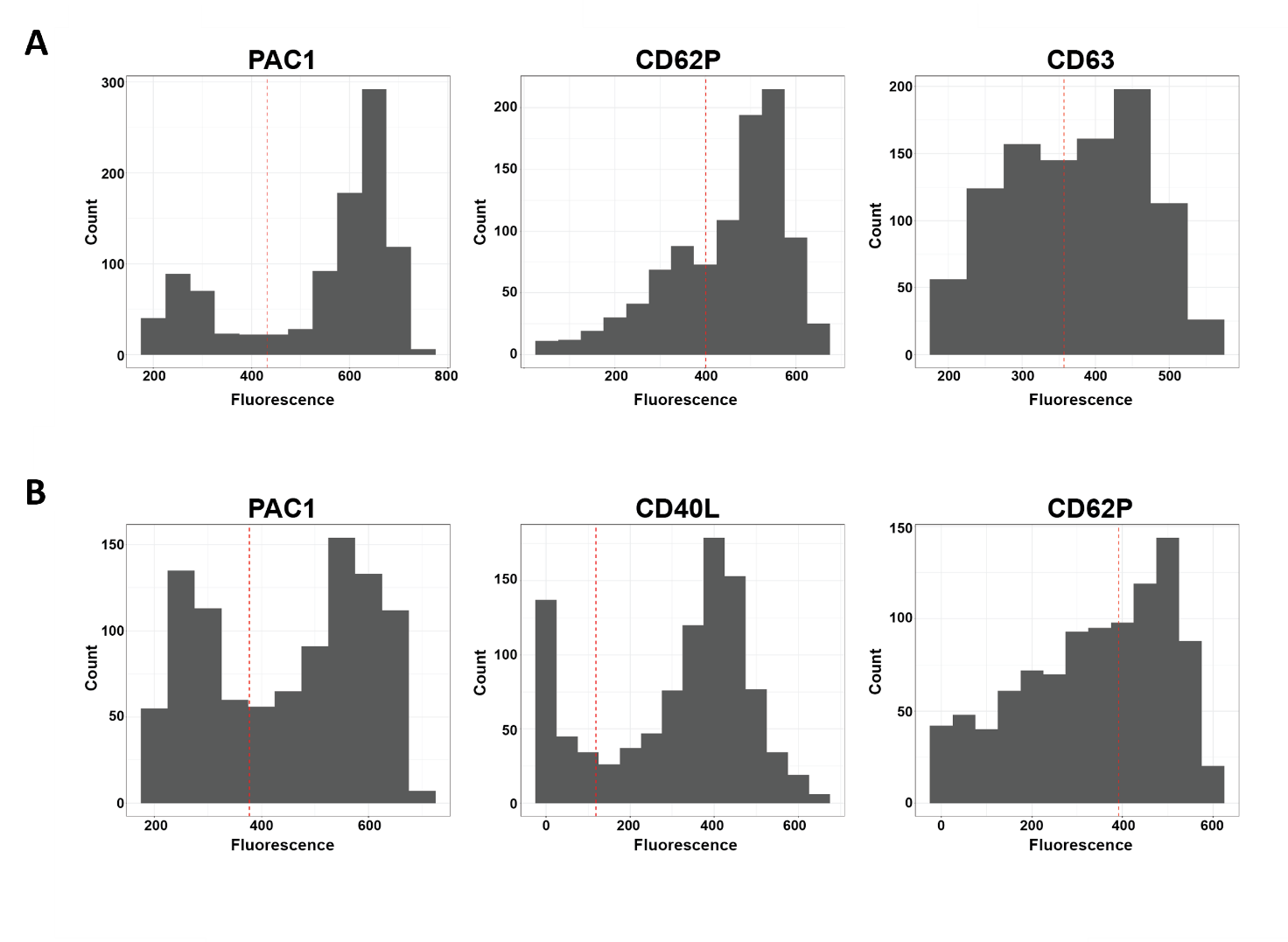
Supplementary Figure 1. Automated thresholds of platelet surface marker fluorescence using FAUST.** Whole blood was unstimulated (basal) or stimulated with ADP (10 µM), TRAP-6 (10 µM) or CVX (2-10 ng/mL) for 20 minutes prior to fixation. Fixed samples were then analysed by flow cytometry where the fluorescence intensity of CD62P, PAC1, CD40L, CD63, CD42b, CD147, CXCR4, CD32a and CD36 was quantified. Platelet subpopulation analysis was then conducted by Full Annotation Using Shape-constrained Trees (FAUST). Markers identified by FAUST as statistically important for subpopulation annotation are highlighted. The red dotted line indicates the fluorescence threshold automatically set by FAUST; fluorescence values to the left of the line are classified as negative (no expression), and values to the right as positive (expression). Panels show: **(A)** markers annotating ASP1-8 and **(B)** markers annotating ISP1-8. Data represent four independent donors.

**
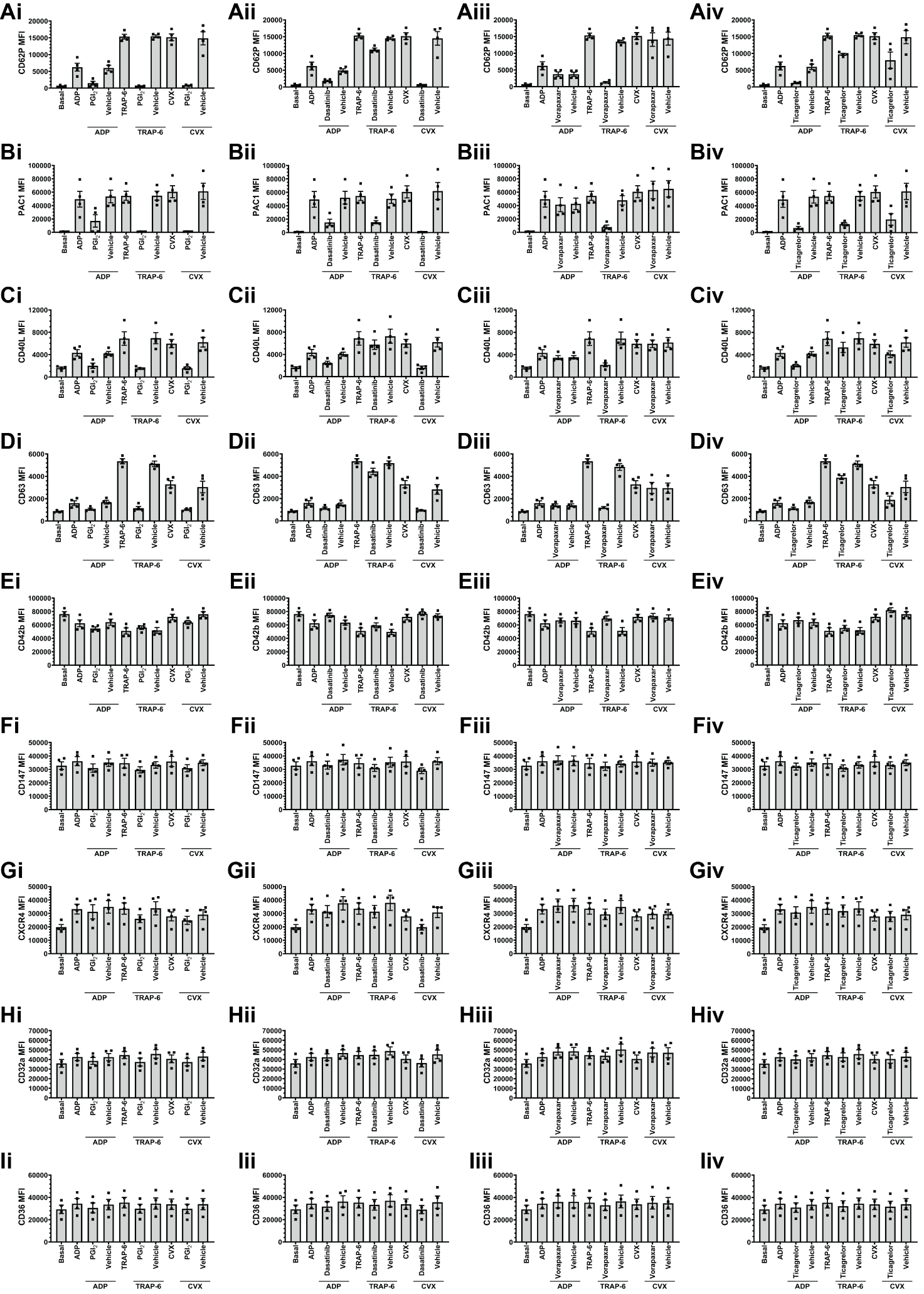
**

**Supplementary Figure 2. The effect of platelet inhibitors on ADP-, TRAP-6-, and CVX-mediated platelet activation and platelet surface markers.** Whole blood (WB) was unstimulated (basal) or stimulated with ADP (10 µM), TRAP-6 (10 µM) or CVX (2 ng/mL) for 20 minutes prior to fixation. When inhibitors were used, WB was pre-treated with the inhibitor (or vehicle control) prior to agonist stimulation. WB was pre-treated with 5 nM PGI_2_ (0.05% ethanol) for 2 minutes, or 500 nM Dasatinib (0.05% DMSO), 1 µM Vorapaxar (0.2% DMSO) or 1 µM Ticagrelor (0.05% ethanol) for 15 minutes. Fixed samples were then analysed by flow cytometry where the median fluorescence intensity (MFI) of **A)** CD62P, **B)** PAC1, **C)** CD40L, **D)** CD63, **E)** CD42b, **F)** CD147, **G)** CXCR4, **H)** CD32a and **I)** CD36 was quantified. Data are expressed as mean ± SEM and were analysed by a repeated measures one-way ANOVA followed by a Šídák multiple comparison test (n=4).

| **Antibody Conjugate** | **Platelet Receptor** | **Antibody Subclass** | **Clone** | **Lot number** | **Laser (nm)** | **Detector (BP)** | **Optimal Titre of Antibody (µg/mL)** |
| --- | --- | --- | --- | --- | --- | --- | --- |
| APC/Cyanine7 Mouse Anti-Human CD42b | CD42b | IgG1, κ | HIP1 | B332984 | 640 | 780-60 | 3 |
| PE/Dazzle™ 594 Mouse Anti-Human CD154 (CD40L) | CD40L | IgG1, κ | 24-31 | B329348 | 561 | 610/20 | 3 |
| PerCP/Cyanine5.5 Mouse Anti-Human CD36 | CD36 | IgG2a, κ | 5-271 | B313700 | 488 | 690/50 | 1 |
| FITC Mouse Anti-Human αIIbβ3 | αIIbβ3 activation | IgM, κ | PAC-1 | B376732 | 488 | 525/40 | 4 |
| PE/Cyanine7 Mouse Anti-Human CD147 | CD147 | IgG1, κ | HIM6 | B295847 | 561 | 780/60 | 3 |
| Alexa Fluor® 700 Mouse Anti-Human CD62P | CD62P | IgG1, κ | AK4 | B336173 | 640 | 712/25 | 1 |
| Brilliant Violet 650™ Mouse Anti-Human CD63 | CD63 | IgG1, κ | H5C6 | B321043 | 405 | 660/20 | 4 |
| Brilliant Violet 421™ Mouse Anti-Human CD184 | CXCR4 | IgG2a, κ | 12G5 | B354131 | 405 | 450/45 | 3 |
| PE Mouse Anti-Human FcγRIIa (CD32) | CD32a | IgG2b, κ | IV3 | 2235902 | 561 | 585/42 | 1 |

**Supplementary Table 1. Antibody information.**

| **Comparison** | **Mean ± SEM / median (IQR)** | **p value** |
| --- | --- | --- |
| **Multiparameter flow cytometry identifies changes in platelet surface markers in response to platelet activation** | | |
| Basal vs 2 µM ADP: CD62P MFI | 1186.00 ± 145.70 vs 5447.00 ± 587.00 | p=0.01 |
| Basal vs 2 µM ADP: CD40L MFI | 2433.00 ± 173.60 vs 5259.00 ± 350.60 | p= 0.01 |
| Basal vs 2 µM ADP: CD63 MFI | 913.60 ± 116.90 vs 1555.00 ± 211.40 | p= 0.03 |
| Basal vs 2 µM ADP: CXCR4 MFI | 20169.00 ± 1444.00 vs 35258.00 ± 2542.00 | p= 0.01 |
| Basal vs 10 µM ADP: CD62P MFI | 1186.00 ± 145.70 vs 7472.00 ± 760.80 | p= 0.01 |
| Basal vs 10 µM ADP: PAC1 MFI | 911.50 ± 228.50 vs 52969.00 ± 7579.00 | p= 0.02 |
| Basal vs 10 µM ADP: CD40L MFI | 2433.00 ± 173.60 vs 6152.00 ± 379.30 | p= 0.003 |
| Basal vs 10 µM ADP: CD63 MFI | 913.60 ± 116.90 vs 1902.00 ± 252.90 | p= 0.02 |
| Basal vs 10 µM ADP: CD147 MFI | 41391.00 ± 1785.00 vs 47481.00 ± 1742.00 | p= 0.03 |
| Basal vs 10 µM ADP: CXCR4 MFI | 20169.00 ± 1444.00 vs 37040.00 ± 2028.00 | p= 0.002 |
| Basal vs 10 µM TRAP-6: CD62P MFI | 1186.00 ± 145.70 vs 19529.00 ± 1948.00 | p= 0.01 |
| Basal vs 10 µM TRAP-6: CD40L MFI | 2433.00 ± 173.60 vs 9794.00 ± 484.50 | p= 0.002 |
| Basal vs 10 µM TRAP-6: CD63 MFI | 913.60 ± 116.90 vs 6356.00 ± 781.90 | p= 0.02 |
| Basal vs 10 µM TRAP-6: CD147 MFI | 41391.00 ± 1785.00 vs 46564.00 ± 1768.00 | p= 0.04 |
| Basal vs 10 µM TRAP-6: CXCR4 MFI | 20169.00 ± 1444.00 vs 35104.00 ± 2206.00 | p= 0.003 |
| Basal vs 10 µM TRAP-6: CD32a MFI | 55532.00 ± 12956.00 vs 71438.00 ± 14225.00 | p= 0.01 |
| Basal vs 2 ng/mL CVX: CD62P MFI | 1186.00 ± 145.70 vs 17188.00 ± 813.30 | p= 0.002 |
| Basal vs 2 ng/mL CVX: PAC1 MFI | 911.50 ± 228.50 vs 73633.00 ± 10320.00 | p= 0.02 |
| Basal vs 2 ng/mL CVX: CD40L MFI | 2433.00 ± 173.60 vs 8464.00 ± 339.20 | p= 0.001 |
| Basal vs 2 ng/mL CVX: CD147 MFI | 41391.00 ± 1785.00 vs 46742.00 ± 2011.00 | p= 0.001 |
| Basal vs 2 ng/mL CVX: CXCR4 MFI | 20169.00 ± 1444.00 vs 30422.00 ± 1252.00 | p= 0.003 |
| Basal vs 10 ng/mL CVX: CD62P MFI | 1186.00 ± 145.70 vs 22838.00 ± 1715.00 | p= 0.003 |
| Basal vs 10 ng/mL CVX: PAC1 MFI | 911.50 ± 228.50 vs 101370.00 ± 3473.00 | p= 0.0003 |
| Basal vs 10 ng/mL CVX: CD40L MFI | 2433.00 ± 173.60 vs 9632.00 ± 881.50 | p= 0.01 |
| Basal vs 10 ng/mL CVX: CD63 MFI | 913.60 ± 116.90 vs 6008.00 ± 384.20 | p= 0.003 |
| Basal vs 10 ng/mL CVX: CD147 MFI | 41391.00 ± 1785.00 vs 45997.00 ± 2081.00 | p= 0.01 |
| Basal vs 10 ng/mL CVX: CXCR4 MFI | 20169.00 ± 1444.00 vs 26300.00 ± 1550.00 | p= 0.004 |
| **Multiparameter flow cytometry identifies distinct platelet subpopulations in response to platelet activation** | | |
| Basal vs ADP: ASP1 abundance | 91.79 ± 2.65 vs 3.83 ± 1.25 | p<0.00001 |
| Basal vs TRAP-6: ASP1 abundance | 91.79 ± 2.65 vs 1.23 ± 0.34 | p<0.00001 |
| Basal vs CVX: ASP1 abundance | 91.79 ± 2.65 vs 0.25 ± 0.05 | p<0.00001 |
| ADP vs TRAP-6: ASP2 abundance | 18.94 ± 3.00 vs 2.23 ± 0.55 | p=0.03 |
| ADP vs CVX: ASP2 abundance | 18.94 ± 3.00 vs 1.16 ± 0.28 | p=0.02 |
| ADP vs TRAP-6: ASP5 abundance | 35.74 ± 3.10 vs 8.84 ± 2.35 | p=0.001 |
| ADP vs CVX: ASP5 abundance | 35.74 ± 3.10 vs 9.56 ± 1.78 | p=0.01 |
| Basal vs ADP: ASP8 abundance | 0.16 ± 0.12 vs 37.68 ± 5.89 | p=0.02 |
| Basal vs TRAP-6: ASP8 abundance | 0.16 ± 0.12 vs 84.49 ± 3.61 | p=0.0001 |
| Basal vs CVX: ASP8 abundance | 0.16 ± 0.12 vs 87.85 ± 2.05 | p<0.00001 |
| ADP vs TRAP-6: ASP8 abundance | 37.68 ± 5.89 vs 84.49 ± 3.61 | p=0.001 |
| ADP vs CVX: ASP8 abundance | 37.68 ± 5.89 vs 87.85 ± 2.05 | p=0.001 |
| ADP vs TRAP-6: CD62P fluorescence in ASP8 | 504.00 (472.00-534.00) vs 544.00 (512.00-577.00) | p<0.0001 |
| ADP vs CVX: CD62P fluorescence in ASP8 | 504.00 (472.00-534.00) vs 553.00 (523.00-585.00) | p<0.0001 |
| ADP vs TRAP-6: PAC1 fluorescence in ASP8 | 643.00 (609.00-672.00) vs 627.00 (586.00-661.00) | p<0.0001 |
| ADP vs CVX: PAC1 fluorescence in ASP8 | 643.00 (609.00-672.00) vs 663.00 (639.00-686.00) | p<0.0001 |
| ADP vs TRAP-6: CD40L fluorescence in ASP8 | 476.00 (442.00-505.00) vs 490.00 (447.00-529.00) | p<0.0001 |
| ADP vs CVX: CD40L fluorescence in ASP8 | 476.00 (442.00-505.00) vs 485.00 (440.00-524.00) | p<0.0001 |
| ADP vs TRAP-6: CD63 fluorescence in ASP8 | 403.00 (379.00-431.00) vs 458.00 (423.00-494.00) | p<0.0001 |
| ADP vs CVX: CD63 fluorescence in ASP8 | 403.00 (379.00-431.00) vs 452.00 (418.00-485.00) | p<0.0001 |
| ADP vs TRAP-6: CD42b fluorescence in ASP8 | 672.00 (638.00-711.00) vs 636.00 (611.00-667.00) | p<0.0001 |
| ADP vs CVX: CD42b fluorescence in ASP8 | 672.00 (638.00-711.00) vs 651.00 (626.00-678.00) | p<0.0001 |
| ADP vs TRAP-6: CD147 fluorescence in ASP8 | 629.00 (599.00-661.00) vs 606.00 (583.00-634.00) | p<0.0001 |
| ADP vs CVX: CD147 fluorescence in ASP8 | 629.00 (599.00-661.00) vs 604.00 (582.00-630.00) | p<0.0001 |
| ADP vs TRAP-6: CXCR4 fluorescence in ASP8 | 606.00 (581.00-632.00) vs 585.00 (564.00-608.00) | p<0.0001 |
| ADP vs CVX: CXCR4 fluorescence in ASP8 | 606.00 (581.00-632.00) vs 562.00 (541.00-584.00) | p<0.0001 |
| ADP vs TRAP-6: CD32a fluorescence in ASP8 | 652.00 (615.00-688.00) vs 635.00 (603.00-669.00) | p<0.0001 |
| ADP vs CVX: CD32a fluorescence in ASP8 | 652.00 (615.00-688.00) vs 629.00 (598.00-663.00) | p<0.0001 |
| ADP vs TRAP-6: CD36 fluorescence in ASP8 | 594.00 (551.00-630.00) vs 575.00 (533.00-612.00) | p<0.0001 |
| ADP vs CVX: CD36 fluorescence in ASP8 | 594.00 (551.00-630.00) vs 571.00 (529.00-607.00) | p<0.0001 |
| **Multiparameter flow cytometry identifies differences in agonist sensitivity to platelet inhibitors** | | |
| ADP vs ADP + PGI_2_: CD36 MFI | 34390.00 ± 4613.00 vs 30598.00 ± 4499.00 | p=0.002 |
| TRAP-6 vs TRAP-6 + PGI_2_: CD62P MFI | 15343.00 ± 710.10 vs 585.00 ± 82.22 | p=0.004 |
| TRAP-6 vs TRAP-6 + PGI_2_: PAC1 MFI | 54604.00 ± 6768.00 vs 785.80 ± 53.66 | p=0.04 |
| TRAP-6 vs TRAP-6 + PGI_2_: CD63 MFI | 5348.00 ± 219.00 vs 1151.00 ± 156.50 | p=0.02 |
| TRAP-6 vs TRAP-6 + PGI_2_: CD32a MFI | 44597.00 ± 36380.00 vs 37363.00 ± 4068.00 | p=0.04 |
| CVX vs CVX + PGI_2_: CD62P MFI | 15169.00 ± 1018.00 vs 801.40 ± 111.60 | p=0.01 |
| CVX vs CVX + PGI_2_: CD63 MFI | 3286.00 ± 326.80 vs 1013.00 ± 66.37 | p=0.04 |
| CVX vs CVX + Dasatinib: CD62P MFI | 15169.00 ± 1018.00 vs 596.00 ± 69.92 | p=0.01 |
| TRAP-6 vs TRAP-6 + Dasatinib: PAC1 MFI | 54604.00 ± 6768.00 vs 15477.00 ± 2344.00 | p=0.04 |
| TRAP-6 vs TRAP-6 + Vorapaxar: CD62P MFI | 15343.00 ± 710.10 vs 9722.00 ± 295.50 | p=0.04 |
| TRAP-6 vs TRAP-6 + Vorapaxar: PAC1 MFI | 54604.00 ± 6768.00 vs 8008.00 ± 2916.00 | p=0.04 |
| TRAP-6 vs TRAP-6 + Vorapaxar: CD63 MFI | 5348.00 ± 219.00 vs 1203.00 ± 92.74 | p=0.01 |
| TRAP-6 vs TRAP-6 + Ticagrelor: PAC1 MFI | 54604.00 ± 6768.00 vs 12557.00 ± 2175.00 | p=0.04 |
| CVX vs CVX + Ticagrelor: PAC1 MFI | 60616.00 ± 9035.00 vs 19676.00 ± 8675.00 | p=0.006 |
| TRAP-6 vs TRAP-6 + Ticagrelor: CD63 MFI | 5348.00 ± 219.00 vs 3876.00 ± 171.70 | p=0.04 |
| CVX vs CVX + Ticagrelor: CD63 MFI | 3286.00 ± 326.80 vs 1884.00 ± 343.40 | p=0.02 |
| ADP vs ADP + Ticagrelor: CD32a MFI | 42558.00 ± 3789.00 vs 40179.00 ± 3838.00 | p=0.001 |
| ADP vs ADP + Ticagrelor: CD36 MFI | 34390.00 ± 4613.00 vs30930.00 ± 4250.00 | p=0.04 |
| TRAP-6 vs TRAP-6 + Ticagrelor: CD36 MFI | 35310.00 ± 4739.00 vs 32237.00 ± 4787.00 | p=0.04 |
| **Multiparameter flow cytometry identifies how platelet inhibitors influence the formation of platelet subpopulations in response to platelet activation** | | |
| ADP vs ADP + Ticagrelor: ISP4 abundance | 0.04 ± 0.012 vs 0.14 ± 0.02 | p=0.013 |
| ADP vs ADP + Dasatinib: ISP5 abundance | 22.88 ± 5.98 vs 54.46 ± 3.03 | p=0.044 |
| Basal vs ADP: ISP8 abundance | 0.38 ± 0.13 vs 64.38 ± 7.48 | p=0.0009 |
| ADP vs ADP + PGI_2_: ISP8 abundance | 64.38 ± 7.48 vs 17.19 ± 9.43 | p=0.0009 |
| ADP vs ADP + Dasatinib: ISP8 abundance | 64.38 ± 7.48 vs 19.40 ± 4.57 | p=0.0004 |
| ADP vs ADP + Vorapaxar: ISP8 abundance | 64.38 ± 7.48 vs 45.35 ± 6.00 | p=0.020 |
| ADP vs ADP + Ticagrelor: ISP8 abundance | 64.38 ± 7.48 vs 12.66 ± 2.85 | p=0.0005 |
| TRAP-6 vs TRAP-6 + Vorapaxar: ISP5 abundance | 3.35 ± 0.49 vs 35.65 ± 4.20 | p=0.016 |
| Basal vs TRAP-6: ISP8 abundance | 0.38 ± 0.13 vs 90.14 ± 1.62 | p<0.00001 |
| TRAP-6 vs TRAP-6 + PGI_2_: ISP8 abundance | 90.14 ± 1.62 vs 0.45 ± 0.24 | p<0.00001 |
| TRAP-6 vs TRAP-6 + Dasatinib: ISP8 abundance | 90.14 ± 1.62 vs 81.99 ± 2.01 | p=0.025 |
| TRAP-6 vs TRAP-6 + Vorapaxar: ISP8 abundance | 90.14 ± 1.62 vs 13.85 ± 3.67 | p=0.0001 |
| TRAP-6 vs TRAP-6 + Ticagrelor: ISP8 abundance | 90.14 ± 1.62 vs 72.84 ± 3.10 | p=0.034 |
| Basal vs CVX: ISP8 abundance | 0.38 ± 0.13 vs 89.36 ± 2.48 | p=0.00001 |
| CVX vs CVX + PGI_2_: ISP8 abundance | 89.36 ± 2.48 vs 2.94 ± 1.56 | p<0.00001 |
| CVX vs CVX + Dasatinib: ISP8 abundance | 89.36 ± 2.48 vs 0.03 ± 0.02 | p=0.00001 |

**Supplementary Table 2. Table of statistics.** Data are expressed as mean ± SEM or median (interquartile range, IQR).
